# Beyond Angiography: Cardiac CT for Planning Complex PCI in Calcified Coronary Lesions

**DOI:** 10.3390/tomography12050069

**Published:** 2026-05-12

**Authors:** Kenji Sadamatsu, Kazumasa Kurogi, Yasuhiro Nakano, Takashi Kajiya

**Affiliations:** 1 Department of Cardiology, Omuta City Hospital 2-19-1 Takarazaka-Machi, Omuta 836-8567, Japan; 2 Department of Cardiovascular Medicine, Miyazaki Prefectural Nobeoka Hospital, Nobeoka 882-0835, Japan; hjtxh139@yahoo.co.jp; 3 Department of Cardiovascular Medicine, Kyushu University Hospital, Fukuoka 812-8582, Japan; nakano.yasuhiro.030@m.kyushu-u.ac.jp; 4 Department of Cardiology, Tenyoukai Central Hospital, Kagoshima 892-0822, Japan; t_kajiya@hotmail.com

**Keywords:** coronary computed tomography angiography, coronary artery calcification, percutaneous coronary intervention, calcium modification, intravascular imaging, chronic total occlusion

## Abstract

Coronary artery calcification, present in 20–30% of percutaneous coronary interventions (PCI), significantly impairs procedural success. Conventional angiography detects calcification in fewer than half of affected cases, while intravascular imaging—though precise—requires lesion crossability that cannot be guaranteed in up to 20% of severely calcified lesions. Cardiac CT (CCT) addresses both constraints by providing comprehensive, three-dimensional calcium characterization before the procedure begins, independent of wire crossability. This review details how specific CCT-derived parameters translate into procedural decisions. Calcium arc, depth, density, and longitudinal distribution each carry distinct implications for device selection: superficial high-density calcium favors atherectomy, while deep concentric patterns are better addressed by intravascular lithotripsy. Validated scoring systems—including the ABCD score—enable objective pre-procedural risk stratification. For chronic total occlusions, bifurcation lesions, ostial stenoses, and very long calcified segments, CCT provides lesion-specific information that supports stepwise strategy selection, equipment preparation, and anticipation of combined modification approaches. Importantly, CCT also identifies anatomical configurations—such as left main bifurcations or tortuous calcified segments—where specific device-related risks warrant particular caution. CCT and intravascular imaging serve complementary roles: CCT defines the strategic framework before the procedure, while intravascular imaging guides real-time execution and optimization. Limitations include operator-dependent interpretation, the absence of standardized protocols for translating calcium morphology into device selection, and the need to validate established Hounsfield unit thresholds in emerging photon-counting CT systems. Prospective randomized evidence comparing CCT-guided and intravascular imaging-guided strategies remains limited but is anticipated from ongoing trials.

## Simple Summary

Calcium deposits in coronary arteries make heart procedures technically demanding, increasing the risk of incomplete stent deployment and long-term complications. Conventional X-ray imaging often fails to detect calcium severity, and catheter-based imaging cannot always reach the most heavily calcified lesions. This review demonstrates how pre-procedural cardiac CT scanning characterizes calcium location, depth, and density before any device is introduced, enabling operators to select appropriate modification tools and anticipate procedural risks. Practical frameworks are presented for challenging anatomical subsets, including blocked arteries, branching vessels, and ostial lesions. These CT-guided strategies may improve procedural safety and outcomes while informing the design of future clinical trials.

## 1. Introduction

Coronary artery calcification represents one of the most challenging obstacles in contemporary percutaneous coronary intervention (PCI), profoundly impacting procedural outcomes. Moderate to severe calcification occurs in 20–30% of PCI cases [1], with higher prevalence in elderly patients, those with chronic kidney disease, and diabetic populations. The clinical consequences are substantial: lower procedural success rates, higher periprocedural complications, and persistently elevated rates of long-term adverse events. Most critically, stent underexpansion—the near-inevitable result of inadequate calcium modification—remains the strongest predictor of both stent thrombosis and in-stent restenosis.

To prevent such complications, precise procedural imaging is essential; however, conventional modalities demonstrate critical constraints. Fluoroscopic angiography is inherently luminographic and systematically underestimates calcification severity (40–50% sensitivity) [2]. While intravascular imaging (IVI) offers superior detection, it is restricted by its intraprocedural timing and an inability to cross severely calcified lesions in up to 20% of cases [3]. Cardiac computed tomography (CCT) addresses these fundamental limitations by providing a comprehensive, non-invasive, three-dimensional (3D) assessment regardless of wire crossability. By evaluating lesion characteristics with detailed calcium morphology before the procedure begins, CCT allows clinicians to transition from reactive management to a proactive interventional strategy.

Emerging evidence supports the clinical value of CCT through validated scoring systems that predict procedural complexity [4,5,6]. The importance of detailed calcium assessment is further highlighted by the ECLIPSE sub-study [7], while the ongoing Precise Procedural Planning (P4) trial is evaluating whether CCT-guided strategies achieve outcomes comparable to intravascular ultrasound (IVUS) guidance. However, despite these advancements and the publication of excellent comprehensive reviews [8,9,10,11], a critical disconnect remains in the literature: the lack of technical detail on how to translate these non-invasive findings into specific, actionable procedural decisions.

The present article addresses this unmet need by synthesizing contemporary evidence from the interventional cardiologist’s perspective. We provide a practical framework detailing how CCT-derived parameters—such as calcium depth, density, and distribution—directly guide the selection of plaque modification devices and procedural strategy to realize true precision medicine in complex PCI.

## 2. Limitations of Conventional Imaging and the CCT Advantage

### 2.1. Fluoroscopic Angiography: The Fundamental Limitation

Fluoroscopic angiography, despite being the procedural standard for PCI guidance, demonstrates critical limitations in detecting and quantifying coronary calcification. Studies employing IVI as a reference standard consistently show angiography fails to detect calcification in over 50% of cases, with a sensitivity and specificity of 50 and 95%, respectively [12]. This systematic underestimation stems from angiography’s 2D luminographic nature—providing only contrast-filled lumen silhouettes without vessel wall or plaque visualization.

Even when calcification is angiographically detected as radiopaque densities, critical determinants of procedural success remain invisible: circumferential calcium extent (arc), thickness, longitudinal distribution, and depth within the vessel wall. These morphological characteristics fundamentally determine lesion response to advanced plaque modification devices (APMD) yet are completely inaccessible to fluoroscopic assessment. This information gap forces operators to rely on indirect indicators and clinical judgment, often resulting in suboptimal equipment selection, inadequate lesion preparation, prolonged procedures with multiple device exchanges, and ultimately compromised clinical outcomes.

### 2.2. Intravascular Imaging: Superior Detection, Critical Constraints

IVUS and optical coherence tomography (OCT) partially address these limitations through high-resolution cross-sectional vessel wall visualization. These technologies offer superior calcium detection approaching 100% sensitivity and enable precise measurement of calcium arc, thickness, and length—parameters directly guiding device selection and assessing modification adequacy. OCT provides exceptional spatial resolution (10–20 μm), allowing detailed superficial calcium characterization, while IVUS penetrates deeper structures, revealing the total vessel architecture.

However, IVI suffers from three inherent constraints limiting its role as the sole imaging modality for calcified lesion assessment:

1. Intraprocedural Timing: IVI provides information only after arterial access, anticoagulation, and guide catheter engagement. This timing precludes pre-procedural strategic planning, equipment preparation, and case triage decisions that could optimize resource utilization and procedural efficiency.
2. Lesion Crossability Limitation: Severely calcified or totally occluded lesions may be uncrossable by imaging catheters in up to 20% of cases, creating a paradox where the most challenging lesions requiring detailed assessment are precisely those that cannot be evaluated.
3. Methodological Limitations in Uncrossability Prediction: The current understanding of calcium morphologies predicting device uncrossability has important methodological limitations. Available evidence derives primarily from retrospective analyses of lesions requiring atherectomy use rather than from dedicated prospective studies specifically examining technical device crossability. An IVUS-based analysis revealed that uncrossability reflected not only severe calcification but also the interaction between calcium morphology and vessel geometry, with the definition depending on operator decision-making patterns [13]. Therefore, CCT-derived predictions of ‘likely uncrossable’ lesions should be interpreted as indicators of high procedural complexity requiring APMD availability, rather than definitive predictions of absolute uncrossability.

### 2.3. CCT: Comprehensive Pre-Procedural Assessment

CCT addresses these fundamental limitations by providing comprehensive, non-invasive 3D coronary visualization prior to intervention—critically, regardless of lesion crossability. Although CCT has lower spatial resolution than IVI, it excels at predicting the difficulty of procedures and evaluating key features for strategic planning.

CCT’s unique advantages include:

- Comprehensive anatomical assessment: Simultaneous evaluation of calcium morphology (arc, length, thickness, density, and distribution), vessel tortuosity, anatomical variants, reference segment characteristics, and myocardial territory at risk
- Pre-procedural strategic planning: Assessment independent of lesion crossability enables advance preparation of appropriate equipment, determination of optimal access approach, realistic procedural time allocation, and informed patient counseling
- Integrated procedural guidance: Identification of optimal fluoroscopic projections, prediction of device deliverability challenges, landing zone evaluation, and objective complexity scoring guiding case selection

### 2.4. Implications for Clinical Trial Design

Recent large-scale trials (ECLIPSE, ROTAXUS) failed to demonstrate superiority of APMD [14,15]. The ECLIPSE OCT substudy revealed why: although 98.1% of lesions met angiographic criteria for severe calcification, less than half were truly severe by OCT (calcium arc ≥ 270°) [16]. This “indication dilution” masked potential benefits in appropriately selected patients.

CCT uniquely addresses this limitation by enabling comprehensive pre-procedural calcium assessment regardless of crossability—a critical advantage since IVI catheters fail to cross severely calcified lesions in up to 20% of cases. CT-based morphological assessment of calcium arc, thickness, length, and distribution can identify patients genuinely requiring advanced modification, potentially revealing true device efficacy in future trials.

## 3. CCT Technology and Image Reconstruction for Interventional Planning

### 3.1. Image Acquisition and Standard Reconstructions

Contemporary CCT protocols utilize high-resolution imaging with sub-millimeter slice thickness, often facilitated by wide-area detector CT that enables single-heartbeat acquisition. To ensure diagnostic quality, particularly in calcified lesions, stringent heart rate control (typically < 60 bpm) via beta-blockade and coronary vasodilation with nitroglycerin is standard. Modern acquisition techniques balance image clarity and radiation dose through prospective ECG-gating or, for patients with high or irregular heart rates, retrospective ECG-gating. While tube voltage is tailored to patient habitus, 120 kVp remains a preferred standard for calcified lesions to ensure sufficient photon penetration and minimize blooming artifacts. Furthermore, the integration of AI-based deep learning reconstruction or advanced iterative algorithms contributes to significant noise reduction. However, their effectiveness in mitigating blooming artifacts is still a subject of discussion; they may not fully overcome inherent spatial resolution limits—even with the advent of ultra-high-resolution CT—when assessing severely calcified segments.

Standard reconstructions include volume rendering and full-volume maximum intensity projection (MIP) for anatomical overview, and curved multiplanar reformation (cMPR) with short-axis views for plaque assessment. While volume-rendering provides intuitive 3D visualization, its utility in severely calcified lesions is limited as calcified shells often obscure the underlying lumen. On the other hand, cMPR straightens the curved vessel into a single plane, which obscures its natural path and orientation. This prevents interventionalists from anticipating vessel overlap or foreshortening, both of which are critical for choosing the best C-arm angles.

### 3.2. Thin-Slab Maximum Intensity Projection: Optimal Technique for Interventional Planning

To overcome these limitations, thin-slab MIP bridges comprehensive CT data with imaging perspectives familiar to interventionalists, preserving original coronary morphology while providing simultaneous anatomical and plaque information. 3D volume data are sectioned at appropriate angles with defined slab thickness—longitudinal views typically use 5 mm thickness, while cross-sectional images use minimum thickness. Operators manually adjust slice angle, depth, and thickness to optimize visualization at specific regions of interest [17].

This technique provides dual perspectives: longitudinal images display vessel morphology, lesion length, and calcium distribution comparable to conventional angiography, while cross-sectional images reveal calcium characteristics analogous to IVI. This side-by-side presentation provides morphological information nearly equivalent to IVI modalities. A key point is that by generating CT images that align with conventional coronary angiography, seamless integration into real-time procedural guidance becomes possible [10].

For patients with renal dysfunction or contrast allergies, thin-slab MIP remains applicable to non-contrast CT. Coronary outlines are identifiable even without contrast, and calcium location can be precisely recognized through angiographic comparison—particularly valuable for heavily calcified lesions when contrast is contraindicated.

### 3.3. Optimization for Calcified Lesion Assessment

Calcified lesion evaluation requires careful window adjustment to minimize blooming artifacts—partial volume effects amplifying apparent calcium size. Window level and width are typically set to 400–600 and 1200–1800, respectively, though optimal values vary with calcium volume [10]. This high window setting is essential for accurate calcium morphology and distribution characterization, which critically determine procedural complexity and device selection strategy.

### 3.4. Future Paradigm: Photon-Counting CT

Photon-counting CT uses semiconductor-based detectors to achieve ultra-high spatial resolution (0.125 mm) and substantially reduces blooming artifacts through improved spatial resolution and spectral energy discrimination [18,19]. These properties offer potential advantages for calcified lesion assessment, including more accurate calcium arc measurement and stenosis quantification in heavily calcified segments. However, photon-counting CT remains unavailable at most institutions, and the Hounsfield unit thresholds used in this review for device selection were derived from conventional CT datasets and will require prospective validation in photon-counting CT acquisitions before direct application. The framework presented here is therefore based on conventional energy-integrating detector CT, which remains the current standard in clinical practice.

## 4. CCT-Based Calcium Characterization and Risk Stratification

Precise characterization of coronary calcification is fundamental to planning complex PCI. Although angiography detects calcification in only 38% of cases [2], CCT provides a comprehensive assessment of calcium morphology, distribution, and density—parameters that critically influence procedural complexity and device selection. A comparison of imaging modalities for the assessment of calcified lesions is summarized in Table 1.

### 4.1. Quantitative Calcium Assessment

#### 4.1.1. Circumferential Extent (Arc)

The calcium arc measured in degrees on cross-sectional images represents the most important procedural difficulty predictor. Categories include mild (<90°), moderate (90–180°), severe (180–270°), and very severe (≥270° circumferential) [20]. CT-derived calcium arc measurements demonstrate excellent correlation with OCT (r = 0.92) and high diagnostic accuracy for detecting severe calcium (arc > 180°: sensitivity 92.4%, specificity 90.9%; arc > 270°: sensitivity 90.3%, specificity 79.7%) [21], with the latter typically requiring APMD [22,23].

#### 4.1.2. Longitudinal Length

The longitudinal length is measured along the vessel axis on longitudinal thin-slab MIP images and/or straight MPR. CT-derived measurements demonstrate excellent agreement with OCT (Pearson’s r = 0.96), confirming the accuracy of the non-invasive calcium length assessment [24]. Extensive calcification (≥5 mm OCT criteria, ≥9 mm CT criteria) is associated with increased complexity, reduced acute gain, and higher stent underexpansion rates, often necessitating multiple balloon inflations or APMD [22,25].

#### 4.1.3. Calcium Density (Hounsfield Units)

Maximum CT density correlates with calcium thickness and resistance to balloon-based therapies. Mean Hounsfield unit (HU) values > 637 have been validated as predictors for requiring rotational atherectomy (RA), serving as objective criteria for pre-procedural device selection [26]. Very high density (>1000 HU) combined with calcium arc > 180° has been proposed as a calcium planning score to help predict the need for calcium modification techniques [27]. Density assessment requires careful technique, typically using regions of interest placed at multiple points along the calcified segment to obtain representative values. Notably, these attenuation thresholds must be interpreted with caution as HU values are energy-dependent; lower tube voltages (e.g., 80–100 kV) significantly increase the values compared to a standard 120 kV setting, potentially affecting the clinical application of fixed cut-offs [28,29].

### 4.2. Qualitative Calcium Morphology

#### 4.2.1. Circumferential Extent Patterns

Beyond quantitative measurement, CCT identifies distinct patterns with prognostic implications. Recent work has established a seven-point morphological classification system ranging from “spot” (≤10% cross-sectional area) to “full moon” calcification (360° arc, 100% cross-sectional area) [30]. The most severe pattern is independently associated with procedural failure and significantly elevated complication rates, with procedural success declining from 93.8% in minimal calcification to 73.3% in full-moon lesions. Proper adjustments in window level and width are mandatory to avoid overestimating calcium extent with default settings. The clinical significance of these patterns varies by lesion location and will be discussed in lesion-specific sections.

#### 4.2.2. Calcium Depth (Distribution Within Vessel Wall)

CCT characterizes the calcium location within the vessel wall, distinguishing superficial (at the luminal surface) from deep (within the vessel wall) calcification, while the spatial resolution is limited. Superficial calcification appears as high-density material directly adjacent to the contrast-filled lumen on cross-sectional images. Deep calcification is identified as calcium deposits separated from the luminal surface by intervening tissue. This distinction provides objective criteria for selecting appropriate plaque modification strategies: superficial calcification favors atherectomy devices, while deep calcification favors intravascular lithotripsy (IVL). However, the differentiation is often unclear, especially in heavily calcified cases, and final device selection should integrate CCT findings with intraprocedural assessment, recognizing that mixed calcium patterns may require sequential or combination strategies.

#### 4.2.3. Longitudinal Distribution Patterns

CCT enables a comprehensive assessment of calcification length along the vessel axis, which is critical for procedural planning. Focal eccentric calcification may be amenable to balloon-based strategies, while diffuse circumferential patterns typically require more aggressive modification approaches. Very long calcified lesions (≥20–25 mm) present unique challenges, including increased procedural complexity, higher technical difficulty, particularly for less experienced operators, and elevated complication rates. Of particular importance, when vessel tortuosity or angulation is present within long calcified segments, the risk of vessel perforation increases substantially. Pre-procedural CCT identification of such anatomy allows operators to anticipate these risks and plan accordingly. In severely calcified long lesions with significant angulation, ‘halfway ablation’ may be considered [31]—restricting calcium modification to the proximal portion while avoiding aggressive ablation through tortuous distal segments.

#### 4.2.4. Calcified Nodules (CNs)

CCNs represent a distinct pathological entity that includes both eruptive calcified nodules (characterized by irregular protruding calcium with disrupted fibrous cap, associated with inflammatory processes and plaque rupture) and non-eruptive calcified nodules (characterized by smooth protruding calcium with intact fibrous cap, arising from pre-existing calcification) [32]. Certain CT features may suggest their presence: protruding calcium morphology on longitudinal views, localized high-density calcium with convex configuration, and associated vessel wall irregularity [33]. Currently, CCT still cannot differentiate between eruptive and non-eruptive subtypes reliably. This limitation reflects not only spatial resolution constraints but also the lack of dedicated clinical studies. However, ongoing advances in CT technology, particularly photon-counting CT with enhanced spatial resolution combined with future dedicated research, hold promise for improved CN characterization and potential subtype differentiation.

### 4.3. CT-Based Predictive Scoring: The ABCD Score Integrates Four Key Morphological Parameters (Figure 1)

Angle (Arc) ≥ 270°, Bifurcation involvement, Calcified mass, Distance ≥ 9 mm (one point each). Score interpretation: 0–1 suggests lower atherectomy probability (consider high-pressure or cutting/scoring balloons); ≥2 indicates high probability (sensitivity 76%, specificity 82.5%) [22]. This provides objective pre-procedural risk stratification guiding equipment preparation, operator selection, and patient counseling—incorporating multiple independent complexity predictors into a simple, reproducible system applicable in routine clinical practice. However, the ABCD score, based on a single retrospective study, requires prospective validation and does not predict specific device selection or combination strategy requirements.

**Figure 1:**
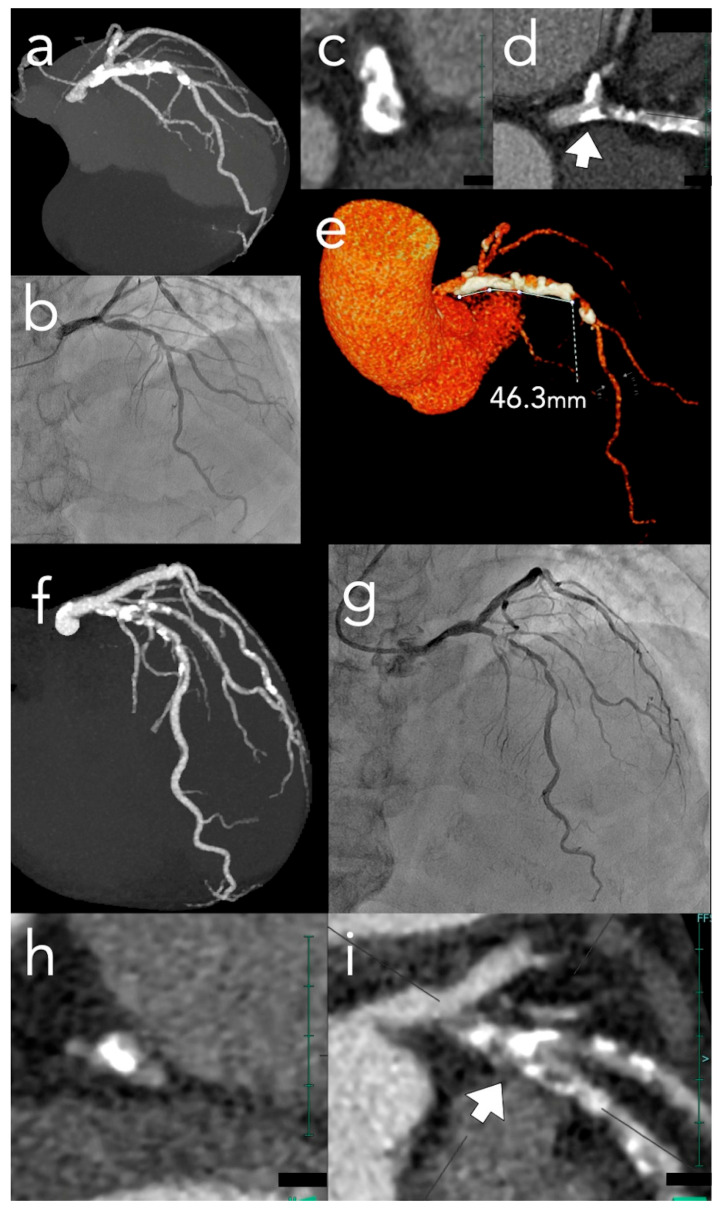
Comparison of the ABCD Score in high- and low-complexity calcified lesions. Upper panels (**a**–**e**): Representative case of a high ABCD score. (**a**) Full-volume maximum intensity projection (MIP) image demonstrates extensive calcification extending from the left main trunk to the mid-left anterior descending artery (LAD), involving the left circumflex ostium. (**b**) Invasive coronary angiography confirms severe calcified stenosis in the ostial-to-proximal LAD. (**c**,**d**) Cross-sectional and longitudinal thin-slab MIP views reveal a circumferential calcification arc ≥ 270° (Angle: 1 point) and bifurcation involvement (Bifurcation: 1 point); the arrow in (d) indicates the level of the cross-section shown in (c). (**e**) Three-dimensional volume-rendered image identifies a calcified segment length of 46.3 mm (Distance: 1 point). With 0 points for Calcified mass, the total ABCD score of 3 predicts a high probability of requiring advanced plaque modification devices. Lower panels (**f**–**i**): Representative case of a low ABCD score. (**f**) Full-volume MIP image shows longitudinal calcification along the proximal-to-mid LAD. (**g**) Invasive angiography confirms the LAD-diagonal bifurcation lesion. (**h**,**i**) Although a calcified bifurcation is identified in the longitudinal thin-slab MIP view (**i**) (Bifurcation: 1 point), the calcification is eccentric with an arc of <180° (Angle: 0 points) and a length of <9 mm (Distance: 0 points); the arrow in (i) indicates the level of the cross-section shown in (h). With 0 points for Calcified mass, the total ABCD score of 1 suggests that conventional balloon-based strategies may be sufficient. ABCD, Angle–Bifurcation–Calcified mass–Distance; LAD, left anterior descending artery; MIP, maximum intensity projection; VR, volume rendering.

### 4.4. Clinical Considerations and Limitations

CCT-based calcium assessment provides comprehensive pre-procedural planning capability as detailed in subsequent sections (Section 5 and Section 6). However, nuanced interpretation is required to account for CCT’s inherent limitations: blooming artifacts often inflate calcium severity, whereas low-density calcium can be obscured by luminal contrast, resulting in potential underestimation [34]. Awareness of these bidirectional diagnostic pitfalls is crucial for optimizing device selection and procedural success.

## 5. CCT-Guided Procedural Planning for Complex Calcified Lesions

### 5.1. General Procedural Planning

**Figure 2:**
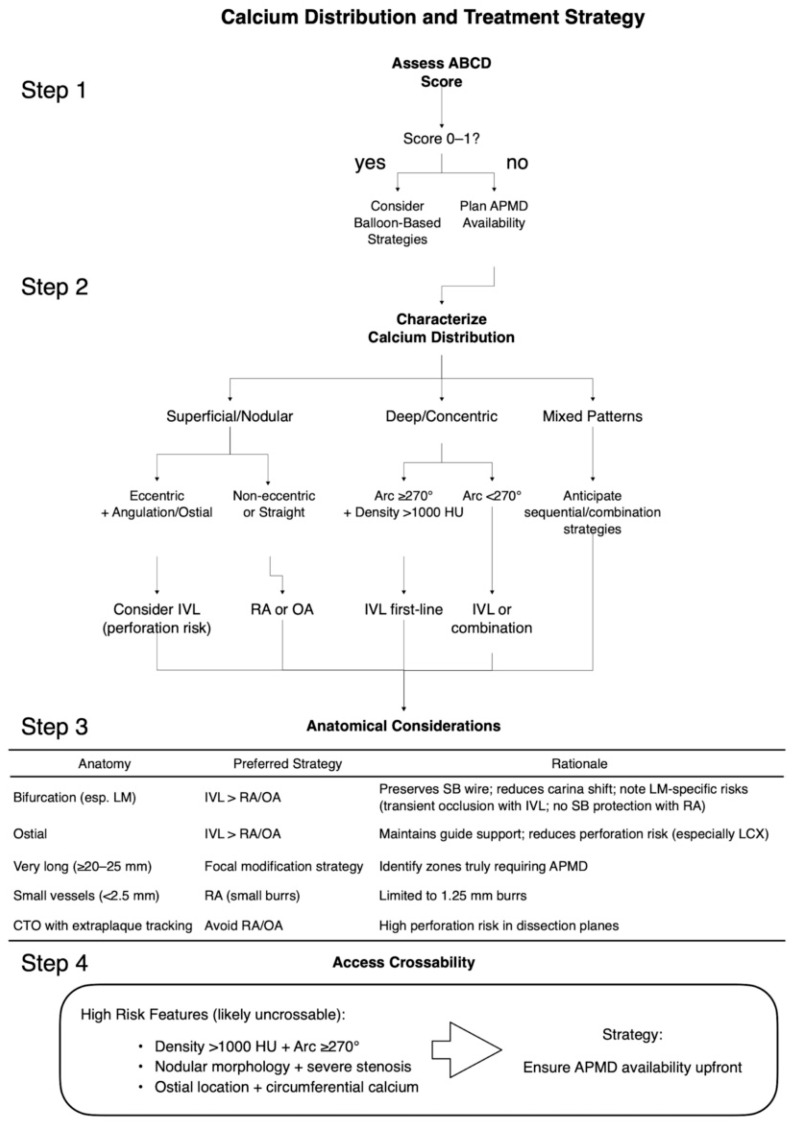
CCT-guided device selection algorithm for severely calcified lesions. A systematic four-step approach integrating ABCD score assessment, calcium distribution characterization, anatomical considerations, and crossability evaluation to optimize device selection and procedural planning. APMD, advanced plaque modification device; CTO, chronic total occlusion; HU, Hounsfield units; IVL, intravascular lithotripsy; LCX, left circumflex artery; LM, left main; RA, rotational atherectomy; OA, orbital atherectomy; SB, side branch.

Beyond general calcium characterization, CCT provides lesion-specific calcium information essential for optimizing procedural strategy in complex subsets where calcification poses particular challenges (Figure 2).

#### 5.1.1. Optimal Fluoroscopic Projection Planning

CCT identifies fluoroscopic angles minimizing foreshortening and overlap—critical in calcified lesions requiring precise device positioning [35]. Three-dimensional reconstruction simulates projections identifying views that clearly separate bifurcations, display full calcified segment length, and optimize landing zone visualization. This pre-procedural angle optimization is particularly valuable in complex calcified lesions where multiple device exchanges may be required.

#### 5.1.2. Landing Zone Assessment and Calcium Management

CCT shows comprehensive pre-procedural evaluation of stent landing zones—critical in calcified lesions where suboptimal selection compromises outcomes. Ideal zones are calcium-free with an adequate diameter; however, diffusely calcified vessels often preclude this option. Superficial calcium arcs of <180° by IVI are generally acceptable for stent deployment [36], and a comparable assessment can be reliably performed using CCT.

CCT-Based Landing Zone Classification:

- Optimal (no calcium or spotty): Prioritize when available.
- Acceptable (mild to moderate calcium < 180° arc, superficial): Usable with careful stent sizing and adequate post-dilation.
- Suboptimal (≥180° arc): Consider focal modification before stenting or alternative stent positioning.

#### 5.1.3. Strategic Planning for Very Long Calcified Lesions

CCT assessment of very long calcified segments (≥20–25 mm) requires careful evaluation beyond simply measuring total calcium length. A critical and often overlooked aspect is determining which portions of the calcified segment truly require APMD. Operators may mistakenly assume that all calcified areas within long lesions need aggressive modification; however, CCT precisely identifies specific zones where calcium morphology (arc ≥ 270°, thickness, nodular configuration) genuinely necessitates modification versus segments where conventional balloon angioplasty may suffice. Notably, short-axis evaluations must account for potential pitfalls: spiral calcium distributions or vessel tortuosity causing axis misalignment may prevent a truly circumferential lesion from appearing as such on a single cross-sectional image. This selective strategy is essential when vessel angulation or tortuosity is present within long calcified segments, as unnecessary modification throughout the entire length increases the risk of perforation.

### 5.2. CCT Calcium Assessment in Chronic Total Occlusions

In chronic total occlusions (CTOs), where conventional angiography provides no information about the occluded segment, the assessment of CCT capabilities is particularly valuable. Moderate-to-severe coronary calcium in CTOs is associated with lower procedural success rates and higher complications. Recent comprehensive reviews have detailed CCT’s multifaceted role in CTO-PCI planning, including AI integration and real-time imaging guidance [37]. This section focuses specifically on calcium characterization within CTOs and its implications for device selection and procedural strategy.

#### 5.2.1. CCT Detection Superiority and Predictive Scoring

CCT provides superior calcium detection (>90% sensitivity vs. ~60% for angiography) [38], enabling comprehensive pre-procedural assessment regardless of occlusion length or crossability. This detection superiority is the primary reason CT-based CTO difficulty scores (CT-RECTOR, CT-derived J-CTO, K-CCT) demonstrate higher predictive accuracy than angiography-based systems [4,5,6].

#### 5.2.2. Proximal Cap Calcium Characterization

A key clinical advantage of CCT is its ability to precisely localize calcium at the proximal cap. When calcification is present, guidewires often deflect into the subintimal space at these sites—a challenge that may persist despite careful manipulation. Pre-procedural identification of calcium distribution via CCT allows operators to anticipate this difficulty and navigate the wire away from calcified areas to minimize the risk of subintimal entry. Furthermore, when subintimal entry is unavoidable, understanding the calcium pattern leads operators to prepare alternative strategies, such as intravascular ultrasound-guided wiring for true lumen visualization or transitioning to a retrograde approach if antegrade attempts are deemed high-risk. A recent study demonstrated that calcification involving ≥50% of the proximal cap cross-sectional area is an independent predictor of failed CTO PCI, with calcium density also significantly associated with procedural failure [39].

#### 5.2.3. Calcium Distribution Within the Occluded Segment

Calcium distribution within the occluded segment impacts both the likelihood of successful wire crossing and procedural strategic planning. CCT-based assessment of calcium patterns predicts the anatomical plane that guidewires are likely to traverse, thereby informing the selection of an optimal wiring strategy (see Appendix A for detailed calcium-based navigation strategies). Specifically, “full-moon” calcification throughout the occlusion is associated with significantly lower success rates (71.4% vs. 87.5%) and higher perforation rates (14.2% vs. 3.5%) [40]. This morphology represents the most severe end of a progressive calcium spectrum; recent evidence demonstrates a dose-dependent relationship therein, where each incremental step in morphology independently predicts longer procedural times, increased fluoroscopic exposure, and greater device utilization [30]. Such severe calcification, often leading to wire crossing times exceeding 30 min and procedural failure, is a key component incorporated into the KCCT score [5]. Similarly, the CT-RECTOR score includes extensive calcification (defined as >50% of the cross-sectional area) as a major predictor of crossing difficulty [6].

Key patterns include:

1. Focal Eccentric or Deep Calcium: When calcium occupies a limited arc and does not extend to the vessel center, or when calcification is predominantly deep, these patterns typically do not impede wire progression. Antegrade wire escalation represents the primary strategy.
2. Deep Circumferential “Guardrail” Calcification: A complete calcific ring within the vessel wall constrains wire movement within the central channel, reducing subintimal entry likelihood and facilitating intimal tracking even in longer occlusions. This pattern indicates confident antegrade approaches.
3. Extensive Superficial Calcium (≥50% cross-sectional area at luminal surface): Wire entry into the subintimal space becomes highly likely due to rigid calcium deflecting the wire away from the true lumen. Operators should either (a) plan bidirectional approaches from the outset or (b) consider antegrade dissection re-entry strategies with tip detection capability.

#### 5.2.4. Practical CT-Angiography Co-Registration for Wire Navigation

When coronary angiography demonstrates visible calcification (~60% of CTO cases), integration of CCT findings with real-time angiographic imaging provides critical strategic advantages. By reconstructing thin-slab MIP images that approximate angiographic projection angles, operators can establish precise anatomical correlation between CT-identified calcium distribution and fluoroscopic landmarks, enabling the prediction of optimal wire passage route through the occluded segment (detailed methodology in Appendix A).

#### 5.2.5. CCT-Guided Strategic Preparation

CCT calcium characterization suggests CTO-specific strategic preparation:

- High-risk calcium patterns (full-moon entry calcification, extensive superficial calcium > 50% cross-sectional area): Anticipate need for APMD or extraplaque approaches; prepare appropriate equipment and allocate extended procedural time; consider bidirectional approaches from the outset.
- Favorable patterns (deep circumferential “guardrail” calcification, focal eccentric calcium): Standard equipment often sufficient; antegrade wire escalation as primary strategy.
- Case selection: CT-based complexity scores guide appropriate matching of case difficulty to operator experience and equipment availability.

Randomized trial data demonstrate that CT-guided CTO PCI achieves higher procedural success rates and fewer complications versus angiography guidance alone [41]. Comprehensive device selection criteria are detailed in Section 6.

### 5.3. Calcified Bifurcation Lesions

CCT provides a detailed 3D assessment of bifurcations, evaluating vessel diameters and angles in addition to calcification morphology. In line with the 18th European Bifurcation Club consensus [42], a stepwise provisional strategy is generally preferred to minimize metal burden. Pre-procedural CCT insights enable a tailored strategy, guiding the selection between Culotte, T and small protrusion, or double-kissing crush based on vessel mismatch, bifurcation angles, and the anticipated risk of side-branch compromise.

#### 5.3.1. Calcium Distribution Assessment

CCT assessment of calcium location at the carina and side branch ostium predicts bifurcation-specific complications. Circumferential calcium (arc ≥ 270°) at these critical locations increases device modification requirements and procedural complexity.

Extensive calcification at the carina creates specific technical challenges: it complicates side branch wire insertion due to the rigid, non-compliant calcium preventing guidewire deflection into the side branch ostium; increases the risk of carina shift during main vessel stent deployment, as calcified plaque resistance to compression can cause unpredictable plaque displacement toward the side branch; and limits the effectiveness of certain bifurcation techniques, such as the jailed balloon technique and kissing balloon inflation, due to inadequate calcium modification. Heavy side-branch ostial calcification may preclude effective intervention, as rigid calcium at the ostium prevents adequate stent expansion even with high-pressure post-dilation.

Calcium burden and distribution are among the most important predictors in established bifurcation scoring systems (CT bifurcation score, RESOLVE score) [43,44]. Specifically, the CT bifurcation score (incorporating side branch plaque, proximal main vessel calcification, low-attenuation plaque, and main vessel/side branch area ratio) offers 90% sensitivity for identifying potential side branch compromise. In parallel, the CT-derived RESOLVE score provides a standardized morphological evaluation that matches the predictive power of invasive angiography, facilitating the identification of high-risk anatomy—such as wider bifurcation angles or significant core stenoses—solely through pre-procedural imaging.

#### 5.3.2. Main Vessel Calcium and Device Selection

IVL is often preferred for bifurcations to preserve side-branch wire access and minimize carina shift risk. Severe carina calcification may necessitate upfront modification before two-stent techniques. In addition, specific technical limitations must be considered in left main bifurcations. Detailed device selection criteria are provided in Section 6 and Table 2.

#### 5.3.3. Landing Zone Assessment: Bifurcation-Specific Considerations

In bifurcations, landing zone assessment requires evaluation of multiple segments:

- Proximal main vessel landing zone: Apply general classification criteria as defined in General Procedural Planning
- Distal main vessel landing zone: Particularly critical when the distal vessel diameter is smaller or has a greater calcium burden
- Side branch ostium (for two-stent techniques): Severe ostial calcification may preclude adequate stent expansion despite aggressive post-dilation, favoring provisional approaches over planned two-stent techniques.

#### 5.3.4. Myocardial Mass at Risk (MMAR) Integration with Calcium Assessment

The ability of CCT to quantify myocardial territory supplied by side branches (MMAR, threshold ≥ 10% for preservation) provides critical context when complex two-stent techniques are particularly challenging due to calcium distribution requiring extensive modification [8,10]. Many anatomically significant side branches supply < 10% MMAR, allowing procedural simplification to provisional approaches compatible with extensive calcium modification strategies [45].

### 5.4. Calcified Ostial Lesions

Ostial lesions complicated by heavy calcification pose unique technical challenges due to their anatomical location and typical circumferential calcium distribution patterns, requiring careful pre-procedural imaging assessment. Particularly, aorto-ostial lesions represent a challenging subset when complicated by heavy calcification because they frequently exhibit circumferential calcification patterns that extend from the vessel ostium into the aortic wall, creating substantial technical challenges for device delivery, adequate lesion preparation, and precise stent positioning [8,46].

#### 5.4.1. Circumferential Extent and Distribution

Cross-sectional images precisely quantify the calcium arc at the ostium. Severe circumferential calcification is particularly common at aorto-ostial locations and predicts APMD need. Importantly, CCT determines whether calcification extends into the aortic wall—a distinction with major implications for guide catheter support and modification strategy.

#### 5.4.2. Calcium Density Assessment

HU measurements guide device selection and predict lesion compliance. Very high-density calcium (>1000 HU) combined with circumferential distribution indicates lesions requiring aggressive modification strategies [27].

#### 5.4.3. Longitudinal Calcium Distribution

An initial assessment of plaque distribution is essential for determining the appropriate stenting strategy.

CCT precisely delineates:

1. Longitudinal extent of calcification: Whether calcium is confined to the ostium or extends into the parent vessel, it guides the decision between precise ostial stenting versus the intentional cross-over technique.
2. Optimal fluoroscopic projection angles: For the ostial stenting approach, CCT identifies angulations that provide perpendicular ostial visualization, critical for accurate stent positioning without protrusion or geographic miss.

Following strategy selection, distal landing zone assessment becomes critical:

- Proximal Landing: Significant protrusion into the parent vessel (aorta or proximal main branch) should be avoided; therefore, precise ostial alignment is mandatory.
- Distal Landing Zone: This site becomes critical as the primary landing zone for ensuring stent stability and long-term patency.

CCT facilitates:

1. Comprehensive assessment of the distal vessel’s calcium burden.
2. Measurement of landing zone length, with a segment of ≥5 mm free of significant calcium typically preferred.
3. Evaluation of the reference vessel diameter for accurate stent sizing.
4. Selection of optimal stent length, balancing complete ostial coverage with landing in less calcified distal segments.

#### 5.4.4. Guide Catheter Support Strategy

Depending on the location of the opening, challenges may arise regarding guide support. By identifying anatomical variations using CCT, it is possible to evaluate support optimization in advance:

- Right coronary artery ostial lesions: CCT visualization of conus branch allows planning for the anchor balloon technique to enhance guide catheter support.
- Left circumflex artery (LCX) ostial lesions: Assessment of LCX-aorta angle and relationship to left main helps anticipate need for alternative guide catheter shapes or support strategies.

#### 5.4.5. CCT-Guided Device Selection Strategy

Ostial-specific considerations (detailed criteria in Section 6 and Table 2):

- IVL preference: Fractures calcium without direct contact, preserves guide position, reduces perforation risk—particularly valuable when calcium extends into the aortic wall or in LCX ostial lesions where atherectomy “burr jump” risk is elevated [47,48,49].
- Atherectomy considerations: If ablative modification is selected, CCT-derived density (>1000 HU) guides smaller burr selection to reduce perforation risk and facilitate controlled ablation.

## 6. CCT-Directed Device Selection Strategy

Management of severely calcified lesions requires effective plaque modification to achieve adequate stent expansion, which remains the most robust predictor of long-term clinical outcomes. CCT-derived calcium characteristics provide objective criteria for device selection (Table 2), facilitating systematic pre-procedural planning that optimizes both equipment availability and procedural efficiency.

### 6.1. CCT-Based Decision Framework

Device selection should integrate CCT-defined calcium morphology with lesion-specific factors. The ABCD score offers an objective risk stratification tool: scores of ≥2 indicate the need for specialized modification devices (e.g., atherectomy), while scores of 0–1 suggest that balloon-based strategies may suffice (Figure 1). Regardless of the target lesion score, the presence of severe proximal calcification may necessitate atherectomy to ensure the deliverability of subsequent devices.

Critically, this precise pre-procedural characterization prevents the underestimation of calcium severity, steering operators away from initially less aggressive balloon-based approaches in high-risk lesions. This is essential because suboptimal ballooning can induce coronary dissections, which are relative contraindications for subsequent rotational or orbital atherectomy, thereby limiting further modification options.

Conversely, a thorough CCT assessment can identify cases where calcification is less challenging than initially suspected. In such selected non-complex lesions, CCT-guided PCI has been shown to be a feasible and efficient alternative to routine IVI, significantly reducing procedural time while achieving excellent stent-sizing accuracy [50].

**Table 2:** CCT-guided device selection criteria for calcified coronary lesions.

Device Type	Primary Indications	CCT-Derived Calcium Characteristics	Anatomical Considerations	Technical Considerations	Relative Contraindications
Cutting/Scoring Balloons [1,51]	• Moderate calcification • Post-APMD adjunctive therapy • Focal lesions	• Arc < 270° • Thickness < 0.5 mm • Focal distribution • Eccentric or concentric rings	• Proximal or focal lesions • Aorto-ostial lesions • Straight segments • After RA/OA/IVL	• Balloon-to-artery ratio 0.8–0.9 • Lower inflation pressure vs. conventional • Multiple blade/element contact	• Severe circumferential calcium (≥270°) • Thick calcium (>0.5 mm) • Severe tortuosity
High/Super-High Pressure Balloons [1,12]	• Crossable undilatable lesions • Stent underexpansion • Failed conventional balloon	• Arc < 270° (non-eccentric) • Moderate density • Length variable	• Non-tortuous segments • Adequate proximal support • Avoid aorto-ostial (geographic miss risk)	• Inflation up to 35–40 atm • Careful sizing to avoid dog-boning • Stiffness limits crossing	• Eccentric calcification • Severe angulation • Distal/tortuous vessels
Rotational Atherectomy [12,49]	• Balloon-uncrossable lesions • Undilatable lesions • Very tight calcified stenoses • Long calcified segments	• Superficial calcium • Nodular calcification • Arc ≥ 270° • High density (>637 HU) • Length ≥ 5 mm (OCT)/≥9 mm (CT)	• Suitable for proximal-mid vessels • Caution in severe tortuosity • Bifurcations (if SB wire removal acceptable) • Small vessels (<2.5 mm with 1.25 mm burr)	• Burr/artery ratio < 0.7 • Speed 135,000–180,000 rpm • Short runs (<15–20 s) • 6F compatible (1.25–1.5 mm burrs) • Wire bias consideration	• Eccentric calcium with severe angulation(perforation risk) • LCX ostial with bending(burr jump risk) • Extraplaque tracking in CTO • Fresh thrombus
Orbital Atherectomy [1,12]	• Undilatable lesions • Superficial/nodular calcium • Alternative to RA	• Superficial calcium • Nodular calcification • Arc ≥ 270° • Concentric or eccentric	• Suitable for straight-moderate tortuous segments • Large vessels (≥2.5 mm preferred) • Bifurcations (if SB wire removal acceptable)	• Bidirectional ablation • Low speed (80,000 rpm) initially • High speed (120,000 rpm) selective • Reduced wire bias vs. RA • 6F compatible	• Severe angulation • Vessels < 2.5 mm (high speed) • Severe tortuosity (high speed) • Fresh thrombus
Intravascular Lithotripsy [12,52,53]	• Deep/concentric calcium • Calcified nodules • Large vessels • Stent underexpansion (off-label) • Bifurcations with SB wire protection	• Deep calcium • Concentric patterns (arc ≥ 270°) • Calcified nodules • Arc ≥ 180° • Thickness variable • Density > 1000 HU (combined with Arc >180°)	• Large vessels (≥2.5 mm) • Bifurcations(preserves SB wire) • Aorto-ostial lesions (preserves guide support) • Eccentric calcium with angulation (safer than atherectomy)	• 1:1 balloon sizing • 4 atm inflation during pulse • Up to 80 pulses (120 with C2+) • Ventricular capture possible • 6F compatible	• Vessels < 2.5 mm • Inability to deliver balloon • Severe proximal tortuosity preventing balloon delivery

APMD, advanced plaque modification devices; CCT, coronary computed tomography; CTO, chronic total occlusion; HU, Hounsfield Unit; IVL, intravascular lithotripsy; LCX, left circumflex artery; OA, orbital atherectomy; RA, rotational atherectomy; SB, side branch.

### 6.2. Device-Specific CCT Selection Criteria

#### 6.2.1. Plaque Scoring and Cutting Balloons

This is referentially indicated for moderately calcified lesions or as an adjunctive therapy following advanced plaque modification to score the calcium surface and facilitate uniform stent expansion. These devices create discrete longitudinal incisions (scoring) that disrupt the continuity of the calcified ring, enhancing vessel compliance. According to the EAPCI and SCAI consensus, a key advantage of these balloons is their ability to achieve effective plaque modification at lower inflation pressures compared to conventional balloons, thereby reducing the risk of unintended vessel trauma and “dog-boning” effects [1,12]. They are particularly effective for eccentric or concentric rings with a thickness of <0.5 mm and for focal lesions where precise device delivery is required.

A critical component of procedural success is optimized sizing based on CCT-derived luminal diameters. Computational evidence suggests that simultaneous contact of multiple blades or scoring elements with the calcified plaque maximizes fracturing stress while minimizing injury to the adjacent normal vessel wall. To achieve this safely, a balloon-to-artery ratio of 0.8 to 0.9 is recommended [51]; CCT allows for the precise selection of an undersized balloon (typically 0.25–0.5 mm smaller than the reference diameter), ensuring effective modification while reducing the risk of coronary perforation associated with oversized balloons. This CCT-guided “conservative sizing and low-pressure expansion” strategy balances procedural safety with predictable plaque modification, making it a versatile tool in the calcified lesion algorithm.

#### 6.2.2. Rotational Atherectomy (RA)

Rotational Atherectomy is particularly indicated for superficial/nodular calcification, balloon-uncrossable lesions, long calcified segments, and high-density calcium. Pre-procedural CT guides burr size selection and approach strategy.

- Critical Considerations: CCT evaluation of vessel geometry within calcified segments is essential—significant bending or tortuosity may necessitate “halfway ablation” strategies. Eccentric calcification in angulated vessels (especially LCX ostial lesions) poses a burr “jump” risk. Operators must assess guidewire position relative to calcium distribution using fluoroscopy and, when available, IVI, as guidewires preferentially track along the outer vessel wall [12]. When wire bias positions the guidewire away from eccentric calcium, alternative strategies must be considered.
- Vessel size limitations: RA is the preferred option for very small vessels (<2.5 mm) as other APMD have minimum vessel size requirements of approximately 2.5 mm, whereas RA can accommodate vessels as small as a 1.5 mm diameter using 1.25 mm burrs.
- Left main bifurcations: A critical technical limitation of RA is the inability to maintain a protective guidewire in the side branch (typically the left circumflex artery). This necessitates careful consideration of the risk of side-branch compromise before proceeding with an ablative strategy in these high-risk anatomical subsets.

#### 6.2.3. Orbital Atherectomy (OA)

Orbital Atherectomy shares similar indications with RA for superficial calcium modification. OA offers theoretical advantages in faster modification through bidirectional ablation and reduced wire bias influence, though direct RA comparative data remain limited. The controlled forward and backward movement allows a flexible approach in tortuous vessels while maintaining effective calcium modification [1].

#### 6.2.4. Intravascular Lithotripsy (IVL)

Intravascular Lithotripsy is referentially indicated for deep and concentric calcification patterns where circumferential shock waves fracture calcium at multiple depths simultaneously. It is particularly effective for calcified nodules (fractures without direct contact), large vessels (≥2.5 mm), bifurcations (preserves side branch wire), ostial lesions (preserves guide support), and eccentric calcification patterns where atherectomy poses an elevated “jump” risk. IVL demonstrates consistent efficacy across concentric, eccentric, and nodular calcium patterns [12]. However, when applying IVL to left main lesions, clinicians must account for the fact that balloon inflation causes temporary but complete occlusion of the left main stem. This may lead to significant hemodynamic implications, particularly in patients with impaired left ventricular function or those with right coronary artery disease that provides collateral flow to the left system. A recent meta-analysis comparing IVL with RA demonstrated that IVL achieves significantly shorter procedural times and reduced contrast use, both particularly advantageous for elderly patients and those with renal dysfunction [52]. Furthermore, the prospective ROLLING STONE registry suggests that IVL is associated with a significantly lower 12-month MACE rate compared with atherectomy (6.8% vs. 14.3%), primarily driven by a reduction in cardiovascular death, although selection bias regarding baseline lesion complexity should be considered [53].

#### 6.2.5. Excimer Laser Coronary Atherectomy (ELCA)

ELCA is a versatile “cold” ablation tool that utilizes photochemical, photothermal, and photomechanical mechanisms to modify plaque. It is particularly effective for thrombus-laden lesions, under-expanded stents, and balloon-resistant calcific plaques, where mechanical atherectomy may be limited [54]. While not currently a primary component of CCT-based algorithms, ELCA provides a valuable bailout or synergistic option, especially when integrated with other plaque modification devices in complex scenarios.

#### 6.2.6. Anticipation of Combined and Bailout Strategies

Detailed characterization of mixed calcium patterns (e.g., simultaneous superficial and deep components) or high-risk features, such as density > 1000 HU combined with a 360° arc, allows for the anticipation of combined or hybrid modification strategies. The definitive decision to employ stepwise or bailout approaches, such as rotational atherectomy followed by IVL, depends on real-time intraprocedural assessment via IVI. However, as recent reviews have systematically described the rationale for these hybrid strategies [55], pre-procedural CCT planning ensures that the required devices are available upfront to address potential single-modality limitations.

### 6.3. Complementary Synergy with Intravascular Imaging

While CCT provides a comprehensive pre-procedural assessment, IVI (IVUS or OCT) remains indispensable for real-time intra-procedural guidance and optimization. The integration of these modalities serves complementary and synergistic decision-making through multi-stage scoring. Pre-procedural risk stratification using the CCT-based ABCD score identifies lesions likely requiring advanced modification, allowing for upfront equipment preparation. Once the procedure begins, IVI-based scoring—such as the revised OCT or IVUS calcium scores [56,57]—validates CCT predictions and provides real-time feedback on the adequacy of plaque modification (e.g., detecting calcium fractures).

Integrated Workflow for Precision PCI:

- Pre-procedural CCT Roadmap: Formulate a primary strategy, including APMD selection (via the ABCD score), anatomical risk assessment, and equipment preparation.
- Angiographic Refinement: Confirm pre-procedural CCT findings and refine the approach using optimal fluoroscopic projections pre-determined by CCT.
- Intra-procedural IVI Validation: If the lesion is crossable, use IVI to validate CCT-predicted calcium characteristics and finalize the choice of lesion preparation.
- Synergistic Lesion Modification: Execute modification based on the combined anatomical insights from CCT and real-time mechanical feedback from IVI.
- IVI-Guided Optimization and Safety Check: Perform final stent sizing and verify optimal expansion, while identifying procedural complications such as edge dissections that may be missed by angiography alone.

### 6.4. Practical Device Selection Algorithm (Figure 2)

Step 1—Assess Calcium Severity:

Apply ABCD score: scores 0–1 consider balloon-based strategies; scores ≥ 2 plan APMD availability.

Step 2—Characterize Calcium Distribution:

Superficial/nodular favor atherectomy (with caution in eccentric patterns); concentric/circumferential favor IVL; eccentric patterns (particularly with angulation or ostial location) assess guidewire position carefully, favor IVL over atherectomy; mixed patterns anticipate combination.

Step 3—Evaluate Lesion-Specific Factors:

- Bifurcation: consider IVL for wire preservation and to avoid carina shift in heavily calcified lesions.
- Very long calcified segments (>25 mm): identify zones where severe calcium morphology genuinely necessitates modification rather than treating the entire length. Particularly when combined with vessel tortuosity or angulation within the calcified segment, consider “halfway ablation” or focal modification strategies (performing atherectomy only in segments truly requiring it) to reduce perforation risk while ensuring adequate preparation for stent delivery.
- Very small vessels (<2.5 mm): limited to RA with small burrs.
- Ostial location: anticipate guide catheter support challenges; for LCX ostial lesions with bending and eccentric calcium, strongly consider IVL over atherectomy due to burr “jump” risk toward carina.

Step 4—Assess Crossability:

Very high density (>1000 HU), circumferential pattern, ostial location, or calcified nodule morphology suggest potential uncrossability; plan calcium modification device availability.

### 6.5. Considerations for Less Invasive Approaches

A detailed CCT calcium assessment can identify cases where apparently severe calcification does not require large-bore systems or extensive modification. Careful 3D evaluation often reveals that concerning calcification is focal, eccentric, or segmented rather than circumferential—patterns potentially manageable with standard techniques through smaller guiding catheters. This “rule-out” capability for truly complex calcium morphology supports appropriate selection of less invasive approaches when safe, avoiding unnecessary equipment escalation while maintaining procedural success [58]. Our recent study supports this approach, showing that CCT-guided PCI in non-complex lesions reduced procedural time from a median of 43 to 25 min while maintaining stent-sizing accuracy comparable to IVUS guidance [50]—findings applicable across healthcare settings with varying interventional capabilities.

### 6.6. Case Selection for Facilities with Limited Device Availability

The comprehensive pre-procedural calcium assessment provided by CCT has particular clinical value for facilities with limited access to APMD. Accurate CCT-based risk stratification optimizes case triage: operators can confidently identify cases suitable for conventional balloon-based techniques, preventing unnecessary treatment deferral. Conversely, when CCT reveals lesion morphology genuinely requiring specialized equipment, facilities can implement planned referral pathways for elective procedures at specialized centers. This proactive approach is substantially safer and more resource-efficient than proceeding with inadequate equipment, which risks procedural failure, complications, and emergency transfers.

## 7. Limitations

The CCT-guided approach has several limitations. First, CCT interpretation and the translation of findings into procedural strategies remain operator-dependent, particularly in assessing complex calcium morphology. The assessment has not been standardized and is significantly influenced by variations in image reconstruction algorithms and window settings. Second, the strategic framework presented in this review is based on conventional energy-integrating detector CT technology; it does not yet incorporate the superior spatial resolution and reduced blooming artifacts offered by emerging photon-counting CT, which are poised to redefine precision imaging in calcified lesions. Finally, while we focus on widely available plaque modification devices, specialized technologies such as ELCA are not yet integrated into our primary CCT-based planning algorithm. This reflects their more specialized clinical indications, as well as our limited institutional experience with this specific modality.

## 8. Conclusions

The interventional management of severely calcified coronary lesions has long been constrained by a fundamental information gap: angiography consistently underestimates calcium severity, while IVI—though precise—requires lesion crossability that cannot be guaranteed in the most complex cases. CCT addresses this gap by providing a comprehensive, 3D calcium assessment before the procedure begins, independent of wire crossability.

This review has detailed how specific CCT-derived parameters translate directly into clinical decisions. Calcium arc, depth, density, and longitudinal distribution each carry distinct procedural implications: superficial high-density calcium favors atherectomy, whereas deep concentric calcification is better addressed by IVL. At the proximal cap of a CTO, calcium occupying more than half the cross-sectional area predicts wire deflection into the subintimal space and should prompt upfront preparation for bidirectional approaches. In bifurcation lesions—particularly those involving the left main—pre-procedural knowledge of carina calcium distribution, vessel diameter matching, and bifurcation angle allows the operator to select between provisional and two-stent strategies before the first wire is placed. Critically, CCT also prevents procedural missteps: identifying high-density circumferential calcium before any balloon attempt steers the operator away from initial strategies that might create dissection and foreclose subsequent atherectomy options.

The relationship between CCT and IVI is one of sequencing, not competition. CCT defines the strategic framework—device selection, fluoroscopic projection planning, landing zone identification, and equipment preparation. IVI then refines and executes that strategy in real time, confirming calcium fracture, verifying stent expansion, and detecting edge dissections beyond CCT’s resolution.

Important limitations must be acknowledged. CCT interpretation remains operator-dependent, and the translation of calcium morphology into procedural strategy is not yet standardized. Several proposed findings for device selection were derived from conventional CT and require prospective validation in photon-counting CT datasets, where blooming artifact reduction fundamentally alters calcium quantification. The P4 trial, currently ongoing, will be the first randomized test of whether CCT-guided strategies match outcomes achieved with IVUS guidance.

## Figures and Tables

**Table 1:** Comparison of imaging modalities for calcified lesion assessment with emphasis on CCT thin-slab MIP technique.

Feature	CCT (Thin-Slab MIP)	Invasive Angiography	IVUS	OCT
General Characteristics				
Timing	Pre-procedural	Intraprocedural	Intraprocedural	Intraprocedural
Invasiveness	Non-invasive	Invasive	Invasive	Invasive
Spatial Resolution	0.2–0.5 mm	0.1 mm	100–200 μm	10–20 μm
Contrast Requirement	Yes (acquisition)	Yes (continuous)	No	Yes (flushing)
Radiation Exposure	Yes (single acquisition)	Yes (continuous)	Yes (fluoroscopy)	Yes (fluoroscopy)
Calcium Detection & Characterization				
Calcium Detection Sensitivity	+++	+	+++	+++
Calcium Arc Measurement	++ (cross-sectional view)	−	+++	+++
Calcium Thickness	++ (blooming artifact)	−	+	+++
Calcium Length	+++ (longitudinal view)	+	+++	+++
Calcium Density (HU)	+++	−	−	−
Calcium Depth Assessment	++	−	++	+++
3D Calcium Visualization	+++	−	+	++
Clinical Utility in Procedural Planning				
Pre-procedural strategy formulation	+++	+	+	−
Predictive assessment of uncrossable lesions	+++	+	−	−
Optimal Fluoroscopic Angle Prediction	+++	−	+	−
Estimation of procedure time and cost	++	+	−	−
Independent of Crossability	+++	+++	− (requires crossing)	− (requires crossing)
Anatomical & Morphological Detail				
Vessel Course & Tortuosity	+++	++	+	+
Bifurcation Morphology	+++	++	++	++
Device Selection Guidance	+++	+	+++	+++
Stent Length Planning	+++	+	+++	+++
Post-Intervention Assessment				
Stent Expansion & Apposition	+	+	+++	+++
Edge Dissection Detection	−	+	++	+++
In-stent Restenosis/Neo-calcification	++	+	+++	+++
Lesion-Specific Applications				
Aorto-ostial Lesions (Protrusion/Angle)	+++	+	++	+
Bifurcation/Side-branch Access	+++	++	++	++
CTO (Course & Stump morphology)	+++	+	+	−
Calcified Nodules	+	−	++	+++
Practical Considerations				
Availability	++	+++	+++	+
Learning Curve	++	+	++	+++
Time Required (per case)	5–10 min (analysis)	Real-time	3–5 min	3–5 min
Cost	++	+	+++	+++

CCT, cardiac computed tomography; CTO, chronic total occlusion; HU, Hounsfield Unit; IVUS, intravascular ultrasound; MIP, maximum intensity projection; OCT, optical coherence tomography. +++ Excellent capability; ++ Good capability; + Fair/Limited capability; − Not applicable/Insufficient capability.

## Data Availability

No new data were created or analyzed in this article. Data sharing is not applicable to this article.

## Abbreviations

The following abbreviations are used in this manuscript:

APMD	advanced plaque modification devices
CCT	cardiac computed tomography
CN	calcified nodule
CTO	chronic total occlusion
HU	Hounsfield unit
IVI	intravascular imaging
IVL	intravascular lithotripsy
IVUS	intravascular ultrasound
LCX	left circumflex artery
MIP	maximum intensity projection
MMAR	myocardial mass at risk
cMPR	curved multiplanar reformation
OA	orbital atherectomy
OCT	optical coherence tomography
PCI	percutaneous coronary intervention
RA	rotational atherectomy

## Appendix A. Detailed CCT-Based Wire Navigation Strategies for Calcified Chronic Total Occlusions

The spatial relationship between calcium and the vessel lumen, as characterized by cardiac computed tomography (CCT), directly informs the anticipated wire trajectory and optimal crossing technique. While recent comprehensive reviews have addressed CCT’s role in CTO-PCI, including scoring systems and general strategic considerations, this supplement focuses on translating detailed calcium morphology into operator-focused, pattern-specific wire navigation strategies. The emphasis is on actionable intraprocedural decisions—specifically, predicting wire trajectory and selecting optimal crossing techniques based on three distinct calcium distribution patterns that fundamentally determine procedural approach.

### Appendix A.1. CT-Angiography Co-Registration

When coronary angiography (CAG) demonstrates visible calcification (approximately 60% of CTO cases), precise integration of CCT and angiographic imaging is achieved through the following steps:

1. Image Reconstruction: Create thin-slab maximum intensity projection (MIP) images using the same projection angles employed during invasive CAG.
2. Anatomical Correlation: Establish precise correspondence between CT-identified calcium distribution and fluoroscopic landmarks visible during the procedure.
3. Wire Route Prediction: Based on calcium location relative to the vessel lumen, predict the optimal wire passage route through the occluded segment.

### Appendix A.2. Detailed Calcium Pattern-Based Navigation Strategies

#### Appendix A.2.1. Pattern 1: Focal Eccentric or Deep Calcium (Not Reaching Vessel Center)

##### Calcium Characteristics

- Limited circumferential arc (<180°).
- Does not extend to the vessel center on cross-sectional imaging.
- Predominantly deep within the vessel wall (separated from the luminal surface by tissue).

##### Wire Trajectory Prediction

When calcium occupies a limited arc and does not extend to the vessel center, or when calcification is predominantly deep within the vessel wall, the wire may navigate around or through the calcium-free portion of the cross-sectional area. The presence of a substantial calcium-free channel allows guidewires to find the path of least resistance toward the distal true lumen.

##### Recommended Strategy

- Primary approach: Antegrade wire escalation.
- Equipment: Standard CTO guidewires, polymer-jacketed wires.
- Technique: Progressive wire stiffness escalation as needed.

##### Intraprocedural Considerations

- Monitor wire position carefully to ensure tracking within the true lumen.
- If resistance is encountered, reassess the wire position before advancing further.
- Consider low-dose contrast injection through a microcatheter to confirm the intraluminal position.

#### Appendix A.2.2. Pattern 2: Deep Circumferential “Guardrail” Calcification

##### Calcium Characteristics

- Circumferential (270–360° arc) deep calcification.
- Separated from the luminal surface by intervening tissue.
- Forms a rigid tubular structure.

##### Wire Trajectory Prediction

This “guardrail” configuration constrains wire movement within the central channel formed by the calcific tube, significantly reducing the likelihood of subintimal entry. The rigid calcific structure acts as a protective boundary, guiding the wire along the intended path even in longer occlusions where wire control is typically more challenging.

##### Recommended Strategy

- Primary approach: Antegrade wire escalation with confidence.
- Equipment: Can proceed with standard equipment.
- Technique:
  − Utilize the guardrail effect to maintain the central wire position.
  − Progressive advancement with standard wire escalation protocol.
  − Less aggressive wire manipulation is required compared to other patterns.

##### Favorable Scenarios

Pattern 2 is particularly advantageous in:

- Longer occlusion lengths (>20 mm), where wire control is typically challenging in non-guardrail patterns.
- Cases where lesion length alone would typically prompt immediate consideration of retrograde or ADR approaches.
- Resource-limited settings where complex equipment availability is restricted.
- Operators with less extensive CTO experience, as the guardrail effect provides inherent wire guidance.

##### Clinical Advantage

Pre-procedural CCT identification of this favorable pattern enables operators to:

1. Proceed confidently with antegrade approaches in lesions that appear complex by length.
2. Avoid unnecessary preparation for complex retrograde or ADR techniques.
3. Optimize procedural efficiency and reduce unnecessary equipment preparation.

#### Appendix A.2.3. Pattern 3: Extensive Superficial Calcium (≥50% Cross-Sectional Area)

##### Calcium Characteristics

- Superficial calcium occupying ≥ 50% of the vessel cross-sectional area.
- Located at or near the luminal surface.
- Creates a rigid, non-compliant barrier at the lumen interface.
- May extend along a significant length of the occluded segment.

##### Wire Trajectory Prediction

When calcium occupies ≥50% of the vessel cross-sectional area at the luminal surface, wire entry into the subintimal space becomes highly likely. The rigid calcium deflects the wire away from the true lumen due to:

1. The “hardness mismatch” between the guidewire tip and calcified plaque.
2. Natural tendency of wires to follow the path of least resistance.
3. Limited ability to penetrate or navigate around extensive superficial calcium.

##### Wire Behavior at Calcium Interface

As the guidewire advances and encounters the extensive superficial calcium, it cannot penetrate the rigid calcified surface. Instead, the wire tip deflects along the calcium surface and typically enters the subintimal space at the junction between calcified and non-calcified segments. This subintimal entry occurs even with careful wire manipulation because the physical properties of the calcium–wire interaction override the operator technique.

##### Recommended Strategies

Option A: Bidirectional Approach (Planned from Outset)

- Rationale: Pre-procedural recognition that antegrade true lumen entry is unlikely.
- Preparation:
  − Arrange dual arterial access from the start.
  − Prepare retrograde equipment (collateral channel selection, specialized wires).
  − Allocate extended procedural time.
- Execution:
  − Simultaneous antegrade and retrograde wire advancement.
  − Utilize a retrograde wire to define the distal true lumen.
  − Consider reverse controlled antegrade and retrograde tracking (CART) or kissing wire techniques.
- Advantages:
  − Higher success rates in challenging anatomy.
  − Reduced radiation and contrast compared to failed antegrade attempts, followed by retrograde.
  − Psychological advantage of the planned comprehensive strategy.

Option B: Antegrade Dissection Re-entry (ADR) with Tip Detection

- Rationale: Accept likely subintimal entry but plan controlled re-entry.
- Requirements:
  − ADR device with tip detection capability (e.g., Stingray system).
  − Pre-procedural CCT identification of potential re-entry zones.
- CCT-Guided Re-entry Planning:
  1. Identify calcium-free zones or gaps in the calcified segment on CCT.
  2. Select a re-entry target where the calcium burden is minimal.
  3. Measure distance from entry point to optimal re-entry zone.
  4. Plan a fluoroscopic projection that clearly displays the re-entry target.
- Execution:
  − Controlled subintimal wire advancement to a predetermined re-entry zone.
  − Position the ADR device at a CCT-identified optimal re-entry point.
  − Confirm position with limited contrast injection if needed.
  − Achieve re-entry in the calcium-free zone identified on pre-procedural CCT.

#### Appendix A.2.4. Important Caveats

Limitation 1: CCT-IVI Discordance:

CCT-derived findings may occasionally differ from intraprocedural intravascular imaging due to:

- Blooming artifacts that may overestimate calcium extent.
- Limited spatial resolution (0.5 mm) compared to OCT (10–20 μm).
- Difficulty differentiating superficial vs. deep calcium in some cases.

Limitation 2: Predictive vs. Definitive:

CCT findings should be utilized as a powerful planning roadmap rather than an infallible predictor. Clinical judgment remains paramount, and operators must be prepared to adapt strategy based on:

- Real-time procedural feedback.
- Actual wire behavior during advancement.
- Intraprocedural intravascular imaging when available.

Limitation 3: Individual Anatomical Variation:

Wire behavior can vary based on factors not fully captured by CCT:

- Exact wire tip position and angle of approach.
- Guidewire characteristics (stiffness, coating, tip shape).
- Plaque composition heterogeneity within calcified segments.

### Appendix A.3. Integration with Intraprocedural Decision-Making

The CCT-based strategies outlined above should be integrated with intraprocedural assessment:

1. Initial Wire Advancement:
  - Attempt the strategy predicted by the CCT calcium pattern.
  - Monitor for expected vs. unexpected wire behavior.
  - Early recognition if the actual behavior deviates from the prediction.
2. Strategy Adjustment:
  - If Pattern 3 calcium but wire achieves true lumen entry: Continue antegrade approach.
  - If Pattern 1/2 calcium, but the wire enters the subintimal space: Switch to ADR or retrograde.
  - Use intravascular imaging to confirm wire position when available.
3. Equipment Preparedness:
  - For Pattern 3: Have retrograde or ADR equipment readily available.
  - For Pattern 1/2: Standard equipment is sufficient, but prepare a backup strategy.
  - All cases: Maintain flexibility to adapt based on procedural findings.
4. Clinical Application Algorithm
  Step 1: Pre-procedural CCT review
  - Classify the calcium pattern at the entry point and throughout occlusion.
  - Identify Pattern 1, 2, or 3 characteristics.
  - Measure calcium arc, thickness, and longitudinal extent.
  - Determine optimal fluoroscopic projections that clearly visualize calcium distribution and wire trajectory.
  Step 2: Strategic planning based on the pattern
  - Pattern 1: Plan antegrade wire escalation.
  - Pattern 2: Plan the antegrade approach with confidence despite occlusion length.
  - Pattern 3: Plan the bidirectional approach or ADR with pre-identified re-entry zones.
  Step 3: Equipment preparation
  - Pattern 1/2: Standard CTO equipment.
  - Pattern 3: Add retrograde equipment or ADR devices; identify collateral channels.
  Step 4: Intraprocedural execution
  - Implement the planned strategy.
  - Monitor for concordance with predicted wire behavior.
  - Adapt based on actual procedural findings.
  Step 5: Strategy modification if needed
  - If prediction is inaccurate: Switch to an alternative approach.
  - Use intravascular imaging to guide decision-making.
  - Maintain procedural safety as the paramount priority.

### Appendix A.4. Summary

CCT-based calcium pattern recognition enables:

1. Prediction of likely wire trajectory through occluded segments.
2. Pre-procedural strategic planning tailored to specific calcium morphology.
3. Appropriate equipment preparation and resource allocation.
4. Identification of optimal re-entry zones for ADR techniques.
5. Enhanced procedural efficiency through proactive rather than reactive approaches.

However, operators must recognize CCT’s limitations and maintain flexibility to adapt strategies based on intraprocedural findings. The aim is to use CCT findings as a practical guide while preserving clinical judgment at each step of the procedure.
